# A randomized, double-blind, placebo-controlled trial investigating the effects of an *Ocimum tenuiflorum* (Holy Basil) extract (Holixer^TM^) on stress, mood, and sleep in adults experiencing stress

**DOI:** 10.3389/fnut.2022.965130

**Published:** 2022-09-02

**Authors:** Adrian L. Lopresti, Stephen J. Smith, Alexandra P. Metse, Peter D. Drummond

**Affiliations:** ^1^Clinical Research Australia, Perth, WA, Australia; ^2^Healthy Ageing Research Centre and Discipline of Psychology, College of Science, Health, Engineering and Education, Murdoch University, Perth, WA, Australia; ^3^School of Health and Behavioural Sciences, University of the Sunshine Coast, Sippy Downs, QLD, Australia; ^4^School of Psychology, University of Newcastle, University Drive, Callaghan, NSW, Australia

**Keywords:** *Ocimum tenuiflorum* (Indian Holy Basil), insomnia, randomized controlled (clinical) trial, sleep, stress

## Abstract

**Background:**

In Ayurveda, *Ocimum tenuiflorum* (Holy Basil) is referred to as “the elixir of life” and is believed to promote longevity and general wellbeing. Although limited, there are clinical trials to suggest *Ocimum tenuiflorum* has anti-stress effects.

**Purpose:**

Examine the effects of a standardized *Ocimum tenuiflorum* extract (Holixer^TM^) on subjective and objective measures of stress and sleep quality in adults experiencing stress.

**Study design:**

Two-arm, parallel-group, 8-week, randomized, double-blind, placebo-controlled trial. Australian and New Zealand Clinical Trials Registry trial registration number ACTRN12621000609853.

**Methods:**

One hundred volunteers aged 18–65 years received either 125 mg of *Ocimum tenuiflorum* twice daily or a placebo. Outcome measures included the Perceived Stress Scale (PSS) (primary outcome measure), Profile of Mood States, Athens Insomnia Scale (AIS), Restorative Sleep Questionnaire, and the Patient-Reported Outcomes Measurement Information System-29. Sleep quality was also assessed using a wrist-worn sleep tracker (Fitbit), and stress changes were examined by measuring between-group differences in hair cortisol and stress responses after exposure to an experiment stress procedure known as the Maastricht Acute Stress Test (MAST).

**Results:**

Compared to the placebo, *Ocimum tenuiflorum* supplementation was associated with greater improvements in PSS (*p* = 0.003) and AIS (*p* = 0.025) scores; and at week 8, concentrations in hair cortisol were also lower (*p* = 0.025). Moreover, *Ocimum tenuiflorum* supplementation was associated with a buffered stress responses after exposure to the MAST as demonstrated by significantly lower concentrations in salivary cortisol (*p* = 0.001), salivary amylase (*p* = 0.001), systolic (*p* = 0.010) and diastolic (*p* = 0.025) blood pressure, and subjective stress ratings (*p* < 0.001). *Ocimum tenuiflorum* supplementation was well-tolerated with no reports of major adverse effects.

**Conclusion:**

The results from this trial suggest that 8 weeks of supplementation with an *Ocimum tenuiflorum* extract (Holixer^TM^) may reduce objective and subjective measures of stress, and improve subjective measures of sleep quality. However, further research using gold-standard objective sleep measures will be required to substantiate the sleep-related findings.

**Clinical trial registration:**

https://www.anzctr.org.au/ACTRN12621000609853p.aspx, identifier: ACTRN12621000609853p.

## Introduction

*Ocimum tenuiflorum*, also known as *Ocimum sanctum*, Holy basil or Tulsi, is an aromatic plant native to south-east Asia that has been commonly used in Indian traditional medicine. In Ayurveda, *Ocimum tenuiflorum* is referred to as “the elixir of life” and is believed to promote longevity (1). *Ocimum tenuiflorum* has been demonstrated through *in vitro*, animal, and clinical trials to have anti-stress, adaptogenic, antioxidant, analgesic, anti-asthmatic, and anti-inflammatory properties (2–4). These beneficial effects are believed to be derived from its many biochemically-active constituents including eugenol, carvacrol, ursolic acid, β-caryophyllene, and rosmarinic acid (1, 2, 5–7).

Clinical trials into the beneficial effects of *Ocimum tenuiflorum* are increasing, and in a 2017 systematic review, 24 studies were identified investigating the effects of *Ocimum tenuiflorum* on metabolic-related disorders such as type 2 diabetes and metabolic syndrome; immunity; and neurocognitive function (8). Based on this review, the authors concluded that the evidence of the therapeutic efficacy of *Ocimum tenuiflorum* was favorable, particularly as an adaptogen that has a role in helping to address the psychological, physiological, immunological, and metabolic stresses of modern living; however, further robust research into the safety and efficacy of *Ocimum tenuiflorum* was required. As an anxiolytic agent, clinical trials are limited, although anti-stress benefits have been identified in adults experiencing stress (9) and generalized anxiety disorder (10) at daily doses of 1,000 and 1,200 mg, respectively. There have been no trials into the sleep-enhancing effects of *Ocimum tenuiflorum*, however, because hyperarousal is postulated to be associated with poor sleep (11), *Ocimum tenuiflorum* may have some beneficial effects on sleep quality. The aims of this study were to validate the potential anti-stress and sleep-enhancing efficacy of a phytochemically-enriched extract optimized with bioactives derived from *Ocimum tenuiflorum* (Holixer^TM^) at a lower dose of 250 mg/day in adults experiencing stress and poor sleep. To help understand the mechanisms associated with *Ocimum tenuiflorum*, in particular its influence on the hypothalamus-pituitary-adrenal (HPA) axis and sympathoadrenal medullary system (SAM), the effects of *Ocimum tenuiflorum* on physiological and subjective responses after exposure to the Maastricht Acute Stress Test (MAST) was conducted. It was hypothesized that *Ocimum tenuiflorum* would reduce self-reported stress, improve sleep quality, and reduce physiological and subjective markers triggered by the MAST.

## Materials and methods

### Study design

**Figure 1:**
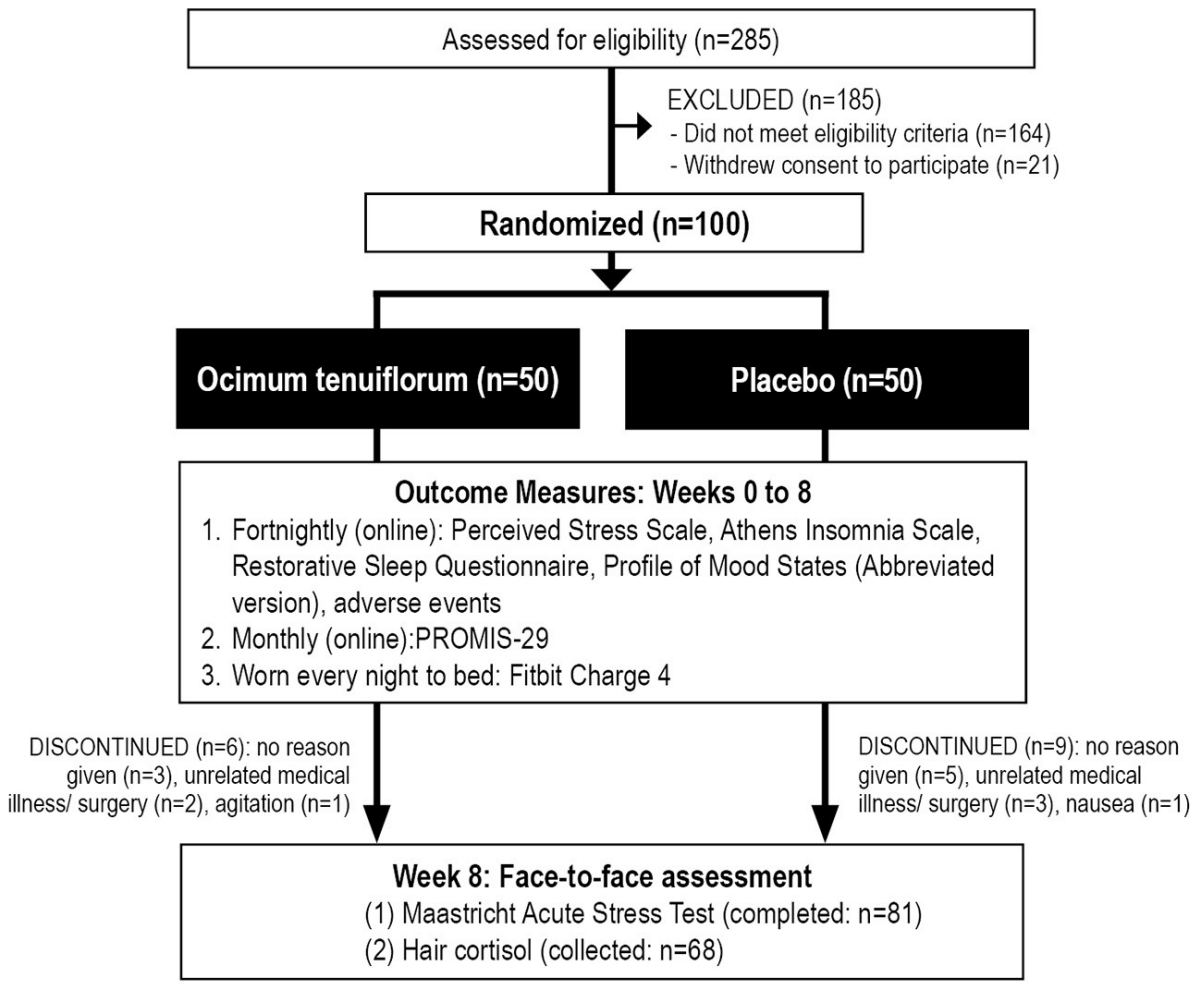
Systematic illustration of study design.

This was a two-arm, parallel-group, 8-week, randomized, double-blind, placebo-controlled trial (Figure 1). All participants gave informed consent, and the trial protocol was approved by the Human Research Ethics Committee at the National Institute of Integrative Medicine (approval number 0086E_2021). This study was prospectively registered with the Australian and New Zealand Clinical Trials Registry (ACTRN12621000609853).

### Recruitment and randomization

Participants were recruited through social media advertisements and email databases between June and September 2021. Interested volunteers visited a website page that provided details about the trial and a link to complete an online screening form that assessed self-reported stress and sleep problems; history of medical or psychiatric disorders; medication use; alcohol, nicotine, and other drug use; and supplement and vitamin intake. To assess the severity of stress, depression, anxiety, and sleep problems, respondents completed the 10-item Perceived Stress Scale (PSS) (12), 4-item Patient Health Questionnaire (PHQ-4) (13) and the Insomnia Symptoms Questionnaire (14). If assessed as likely eligible (see “Participants” section, below), volunteers participated in a telephone interview where they were asked a series of questions to confirm their eligibility and to obtain further demographic details. Suitable participants were then required to complete further online questionnaires comprising the Profile of Mood States, Abbreviated Version (POMS-A), Patient-Reported Outcomes Measurement Information System−29 (PROMIS-29), Athens Insomnia Scale (AIS), the Restorative Sleep Questionnaire, weekly version (RSQ-W), and an online consent form.

Eligible and consenting participants were then randomly allocated to one of two groups (*Ocimum tenuiflorum* or placebo; 1:1 ratio). To ensure sequence concealment, a randomization calculator (http://www.randomization.com) was used with the randomization structure comprising 10 randomly permuted blocks, with 10 participants per block. The randomization sequence was completed by a researcher not directly involved in participant recruitment, and details of bottle codes were held by the study sponsor and revealed after all data collection. Identification numbers were assigned to participants based on their order of enrolment in the study. All capsules were packaged in identical bottles labeled by two intervention codes (held by the sponsor until all data was collected). Study investigators and participants were blind to the treatment group allocation until all outcome data were collected. Participants were posted a Fitbit Charge 4 and their capsule bottles.

### Participants

#### Inclusion criteria

Male and female participants aged 18 to 65 years, experiencing stress for longer than a month, and currently experiencing sleep problems (either difficulty falling asleep, staying asleep, or unrefreshing sleep). Participants were required to score 14 or higher on the PSS and give a rating of 3 or higher on at least one of the first 5 questions of the ISQ. Volunteers had a body mass index (BMI) between 18.5 and 35 kg/m^2^, reported no plan to start new treatments during the study period, were medication free for at least 3 months, apart from the use of analgesics (no more than once a week) or the contraceptive pill, were non-smokers, fluent in English, and willing to wear a Fitbit to bed throughout the duration of the trial. All participants consented (*via* an online consent form) to all pertinent aspects of the trial.

#### Exclusion criteria

Participants were considered ineligible if they anticipated experiencing a major lifestyle change or medical procedure during the study period, were diagnosed with a serious psychiatric disorder or scored >8 on the PHQ-4 (indicating moderate-to-severe depression and/or anxiety), or suffered from medical conditions including but not limited to: diabetes; hyper- or hypotension; cardiovascular disease; a gastrointestinal disease requiring regular use of medications; gallbladder disease; autoimmune disease; endocrine disease; and acute or chronic pain condition. Individuals consuming more than 4 cups of coffee (or equivalent caffeine levels from other caffeinated beverages) or taking herbal or vitamin supplements that were expected to significantly influence study outcomes were ineligible to participate in the study. Moreover, a current or 12 month history of illicit drug use, alcohol intake >14 standard drinks per week, pregnant women, women who were breastfeeding, or women who intended to fall pregnant were also ineligible for the study.

### Interventions

The intervention containing an *Ocimum tenuiflorum* extract (Holixer^TM^) was supplied by Natural Remedies, Bangalore, India. Holixer^TM^ is a patent-pending (202241023540) hydroalcoholic extract derived from the leaves' rich aerial parts of *Ocimum tenuiflorum*. It is manufactured in a good manufacturing practice (GMP)-approved facility and is phytochemically standardized to ≥ 5%w/w (by High-Performance Liquid Chromatography) of the Ocimum Bioactive Complex. It complies with the United States Pharmacopeia (USP) requirements on microbial, heavy metals, residual pesticides, aflatoxins and residual solvents limits for dietary supplements.

Ocimum and placebo capsules were identical in appearance, being matched for color, shape, size, smell, and taste. The intervention contained 62.5 mg of *Ocimum tenuiflorum* extract per capsule. The placebo capsules contained microcrystalline cellulose and syloid. Both intervention and placebo were produced in a GMP–certified facility. All participants were instructed to take 2 capsules, twice daily with or without food for 8 weeks, delivering 250 mg daily of *Ocimum tenuiflorum*. Capsule adherence was evaluated by asking participants to estimate the consistency of capsule intake (0 to 100%), record the intake of morning and evening capsule intake on a mobile phone pill reminder/ monitoring application, and the return of unused capsules at the final assessment. Treatment blinding was evaluated by asking participants to predict group allocation (placebo, *Ocimum tenuiflorum*, or unsure) at the end of the study.

### Outcome measures

#### Primary outcome measure

##### Perceived stress scale

The PSS is a widely used and validated self-report questionnaire measuring the degree to which situations in one's life are evaluated as stressful over the last month (12). Scores on the PSS range from 0 to 40, with higher scores indicating higher perceived stress. An online version of the PSS was completed fortnightly.

#### Secondary outcome measures

##### Profile of mood states, abbreviated version

The 40-item POMS-A is a psychometrically-validated self-report questionnaire that assesses a respondent's current mood state (15). Questions are rated on a 4-point scale (not at all to extremely), and scores are calculated for tension, anger, fatigue, depression, esteem-related affect, vigor, confusion, and total mood disturbance. The POMS-A was completed fortnightly.

##### Athens insomnia scale

The AIS is a validated 8-item scale that assesses the severity of insomnia (16). The questions evaluate the areas of sleep onset, night and early-morning waking, sleepiness during the day time, sleep time, sleep quality, duration and frequency of complaints, distress caused by the experience of insomnia, and interference with daily functioning. The AIS was completed fortnightly.

##### Restorative sleep questionnaire, weekly version

The RSQ-W is a validated 11-item questionnaire that assesses restorative sleep by asking respondents to rate on a 5-point scale feelings of tiredness, mood, and energy (17). The RSQ-W was completed fortnightly.

##### Patient-reported outcomes measurement information system

The PROMIS is a validated, health-related quality of life self-report questionnaire. Scores are calculated for: (1) Physical function, (2) Anxiety, (3) Depression, (4) Fatigue, (5) Sleep disturbance, and (6) the ability to participate in social roles and activities (18). Scores on the physical function and social roles subscales were reverse-scored so that higher scores on all subscales indicate an improvement in symptoms. The PROMIS-29 was completed monthly.

##### Sleep changes as measured by a Fitbit (Fitbit Inc., San Francisco California)

All participants wore a Fitbit Charge 4 to bed every evening. Mean weekly measurements (weeks 1 to 8) in total sleep time, sleep efficiency, and time in bed were calculated. The information collected from Fitbit devices is comparable to data collected by actigraphy and sleep diaries but tends to overestimate total sleep time and sleep efficiency compared to polysomnography (19–21).

##### Maastricht acute stress test

The MAST is a validated experimental stress procedure that has been widely used as a safe and reliable measure of people's response to stress (22, 23). During the 12 min MAST, participants were required to place their hand in cold water at 7°C (Thermoline, TWBRC-12–Refrigerated Waterbath) and complete an arithmetic task (counting backward by 7s) for alternating time intervals. Blood pressure was measured at 7 time points (before MAST instructions, immediately after MAST instructions, immediately after MAST, and 10, 20, 30, and 40 min later). A visual analog scale (VAS) ranging from not at all stressed to extremely stressed, collected before the MAST instructions, immediately after the MAST, and 10, 20, 30, and 40 min later was used to assess changes in self-reported stress over time. Saliva was collected to measure cortisol and α-amylase before the MAST instructions, immediately after the MAST, and 10, 30, and 40 min later. Participants were provided with small collection tubes, and whole saliva was collected by unstimulated passive drool and immediately frozen in a−20°C freezer for later analysis. The MAST was conducted at week 8 by a researcher blinded to group allocation, and for all participants, the procedure was conducted in the same room. On the day of analysis, samples were thawed and precipitated mucins and particulate debris were separated by centrifugation at 1,500 g for 25 min at room temperature. An aliquot of the supernatant was used for the measurement of cortisol and sAA activity. Salivary cortisol was measured using a commercially available competitive cortisol enzyme immunoassay kit (Salimetrics, Carlsbad, CA) according to the manufacturer's instructions and sAA was measured utilizing a kinetic enzyme assay kit (Salimetrics, Carlsbad, CA) according to the manufacturer's instructions.

##### Hair cortisol

Hair provides a chronic measure of free, unbound cortisol (24, 25). In participants with enough scalp hair, a sample of hair was collected by a researcher at the end of the study (week 8) for cortisol analysis. A lock of hair approximately 1–2 mm in diameter was collected from the posterior vertex by cutting as close to the scalp as possible using a pair of fine scissors. Each hair sample was washed 3 x 5 min in methanol to remove any external cortisol on the hair shaft. The hair was left to dry and finely minced with fine surgical scissors before weighing 50 mg for extraction in 1.5 mL methanol for 48 h at room temperature. An aliquot of the methanol was removed and dried under a gentle stream of nitrogen gas at 50°C. The remaining residue was reconstituted in cortisol assay buffer, and centrifuged at 3,000 g to pellet any particulate matter before cortisol determination.

##### Adverse events

The tolerability of capsule intake was assessed every 2 weeks *via* an online question enquiring about adverse effects that were believed to be associated with capsule intake. Participants were also asked to contact researchers if they experienced any adverse effects.

### Sample size calculation

Based on a single outcome variable, an *a priori* power analysis was undertaken to estimate the required sample size. In a previous trial examining the stress-relieving effects of *Ocimum tenuiflorum* in adults experiencing stress-related symptoms, an effect size of 0.8 compared to the placebo was identified (9). As we utilized more robust outcome measures, we predicted a more conservative effect size of 0.6. Assuming a power of 80% and a type one error rate (alpha) of 5%, the number of participants needed per group to find a treatment effect was estimated to be 36. After allowing for an approximate 20% dropout rate, we aimed to recruit 50 participants per group.

### Statistical analysis

For baseline data, an independent samples *t*-test was used to compare group data for continuous variables, and a Pearson's Chi-square test was used to compare categorical data. Outcome analyses were conducted using intention-to-treat (ITT) principles, with all participants retained in originally assigned groups. Generalized Linear Mixed Models (GLMM) assessed for differences between interventions groups on primary and secondary outcomes over time, with intervention effects assessed *via* entry of intervention group (placebo and *Ocimum tenuiflorum)* x time interaction. Time points considered each measure: PSS (primary outcome measure), AIS, RSQ-W, POMS total mood disturbance scores (weeks 0, 2, 4, 6, and 8); PROMIS subscale scores (weeks 0, 4, and 8); and Fitbit measures (weeks 1 to 8). Random intercepts were utilized in each model, and covariates age, sex, and BMI were included. Where applicable, gamma (with log link function) and normal (with identity link function) target distributions were used. Diagonal covariance structures were used to model correlation associated with repeated time measurements in gamma and linear models. Robust estimations were used to handle any violations of model assumptions. Intervention group differences at time points were assessed using simple effects.

Fitbit data were analyzed for each participant with adjustments for artifacts and missing data. This included the removal of daytime naps and the merging of two or more data points if collected during a single overnight sleep. From daily records, mean weekly scores from weeks 1 to 8 were calculated. Between-group differences in changes in MAST measures (systolic and diastolic blood pressure, pulse, VAS ratings, salivary cortisol, salivary sAA) were analyzed using a GLMM (main effects for group and a time x group interaction). Random intercepts were utilized in each model, and covariates were included for age, sex, and BMI. Between-group differences in hair cortisol were analyzed using a GLMM, utilizing random intercepts and the inclusion of age, sex, and BMI as covariates. Pre-treatment concentrations in hair cortisol were originally postulated to be measured in hair 2 to 4 cms from the scalp, and hair 0 to 2 cms from the scalp was theorized to represent cortisol output during the 8-week treatment period. However, as there were significant between-group differences in hair cortisol concentrations during the postulated pre-treatment period (2 to 4 cms from the scalp), only between-group differences of cortisol in newly-acquired hair (0–2 cms), which represented hair cortisol output during the treatment period, was analyzed using a GLLM. A review of the literature suggests that the commonly proposed mean hair growth of 1 cm per month may be an oversimplification as not all hair grows at any one time, and the percentage of follicles in a growth phase is influenced by the anatomical location, age, sex, and race (26–28). All data were analyzed using SPSS (version 28; IBM, Armonk, NY) and the critical *p*-value was set at *p* ≤ 0.05 for all analyses.

## Results

### Study population

#### Baseline questionnaire and demographic information

As detailed in Figure 1, from 285 people who completed the initial online screening questionnaire, 164 individuals did not meet the eligibility criteria and 21 individuals withdrew consent to participate in the study. Online self-report questionnaires from baseline to week 8 were collected from 85 participants, the MAST was completed by 81 participants (4 people either did not attend the final appointment or refused to complete the MAST), and hair samples were collected from 68 participants. Fitbit data was collected from 83 participants, with full nightly sleep data collected from 71 participants. Details of participant background information and baseline scores of the total recruited sample are detailed in Table 1. There were no statistically significant, between-group differences in baseline data. Fifteen participants withdrew from the study, 9 in the placebo group and 6 in the Ocimum group. Reasons for withdrawal included no reason given (*n* = 8), medical illness/surgery unrelated to capsule intake (*n* = 5), increased agitation (*n* = 1), and nausea (*n* = 1).

**Table 1:** Baseline sociodemographic and clinical characteristics.

		**Placebo (*n* = 50)**	***Ocimum tenuiflorum*** **(*n* = 50)**	***p*** **-value**
Age	Mean	44.16	45.96	0.442^a^
	SE	1.72	1.57	
Sex	Female (*n*)	30	34	0.405^b^
	Male (*n*)	20	16	
BMI	Mean	26.08	26.49	0.595^a^
	SE	0.49	0.59	
Marital status	Single	20	19	0.838^b^
	Married/ defacto	30	31	
Educational level	Secondary	24	22	0.840^b^
	Tertiary	15	18	
	Post-graduate	10	10	
IPAC category	Low	28	25	0.703^b^
	Moderate	16	20	
	High	6	5	
PSS	Mean	22.16	21.78	0.701^a^
	SE	0.74	0.66	
AIS	Mean	12.14	11.34	0.946^a^
	SE	0.51	0.43	
RSQ	Mean	37.72	38.11	0.230^a^
	SE	1.91	2.01	
POMS-A Total mood	Mean	117.22	111.72	0.213^a^
disturbance	SE	3.74	2.28	
PROMIS-29 Physical	Mean	4.94	4.68	0.402^a^
function	SE	0.22	0.22	
PROMIS-29 Anxiety	Mean	9.24	0.47	0.342^a^
	SE	0.47	0.38	
PROMIS-29 Depression	Mean	7.48	6.62	0.190^a^
	SE	0.47	0.45	
PROMIS-29 Fatigue	Mean	13.28	12.92	0.622^a^
	SE	0.55	0.48	
PROMIS-29 Sleep disturbances	Mean	14.80	14.68	0.834^a^
	SE	0.43	0.37	
PROMIS-29 Ability to	Mean	10.02	10.34	0.610^a^
participate in social	SE	0.43	0.46	
roles and activities	SE	0.32	0.33	
Total sleep time (Fitbit)	Mean	416.99	417.26	0.980^a^
(min)	SE	8.81	6.10	
Time in bed (Fitbit)	Mean	478.18	478.98	0.902^a^
(min)	SE	9.28	7.01	
Sleep efficiency (Fitbit)	Mean	87.49	87.39	0.944^a^
(%)	SE	0.70	0.52	

a independent samples t-test,

b chi-square analysis.

### Outcome measures

#### Primary outcome measure

##### Perceived stress scale

**Figure 2:**
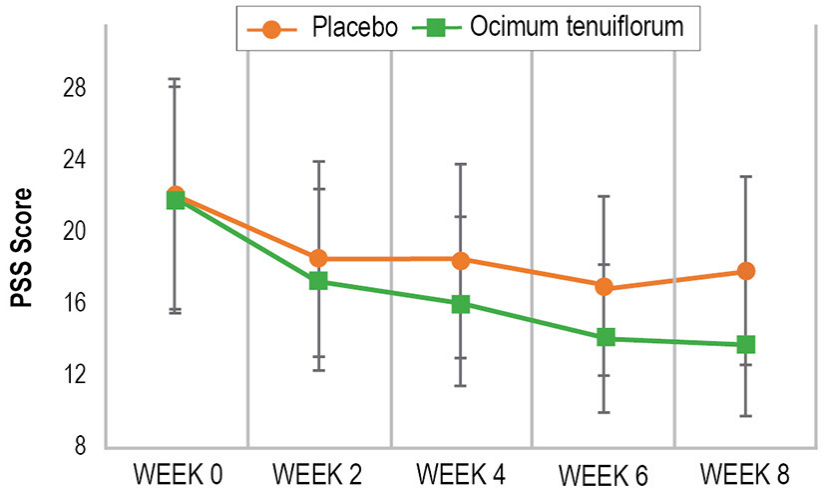
Change in PSS scores over time.

As demonstrated in Table 2 and Figure 2, based on the GLMM, there was a statistically-significant time x group interaction in PSS scores over time (*p* = 0.003). In the Ocimum group, there was a statistically significant 37% decrease in PSS scores from baseline to week 8 (*p* = 0.001) compared to a statistically significant 19% decrease in the placebo group (*p* = 0.004). Moreover, from baseline to week 8, there was a statistically significant greater reduction in PSS scores in the Ocimum group compared to the placebo group (*p* = 0.012). In the Ocimum group, PSS scores reduced by an average of 8.02 points (95% CI, 3.27–12.78, *p* = 001) and in the placebo group, scores reduced by an average of 4.28 points (95% CI, 1.35–7.21, *p* = 0.004). Pairwise contrasts revealed that the between-group differences in change in PSS scores reached statistical significance from week 6 (*p* = 0.047).

**Table 2:** Change in self-report questionnaires (estimated means).

		**Placebo (*****n*** = **41)**						***Ocimum tenuiflorum*** **(*n* = 44)**						***p*** **-value^b^**
		**Week 0**	**Week 2**	**Week 4**	**Week 6**	**Week 8**	**p-value^a^**	**Week 0**	**Week 2**	**Week 4**	**Week 6**	**Week 8**	***p*** **-value^a^**	
PSS	Mean	22.08	18.48	18.36	16.97	17.80	0.004	21.74	17.28	16.09	14.00	13.72	0.001	0.003
	SE	6.47	5.44	5.47	5.19	5.30		6.38	5.09	4.75	4.08	3.97		
AIS	Mean	12.15	8.36	8.80	8.52	8.91	0.028	11.29	8.10	7.57	6.58	5.87	0.010	0.025
	SE	4.51	3.12	3.30	3.21	3.37		4.20	3.03	2.84	2.48	2.21		
RSQ	Mean	37.51	48.59	52.07	51.79	57.00	0.003	37.96	48.11	52.72	54.43	58.15	0.002	0.984
	SE	10.91	14.11	15.11	15.10	16.58		11.05	13.97	15.29	15.84	16.88		
POMS-Total mood disturbance	Mean	117.61	113.87	109.36	107.06	97.30	<0.001	111.86	104.46	99.82	96.26	94.53	<0.001	0.695
	SE	17.20	16.70	16.13	15.85	14.28		16.36	15.32	14.71	14.22	13.83		
PROMIS-physical function	Mean	5.00		5.42		4.98	0.970	4.68		4.76		4.55	0.668	0.800
	SE	0.98		1.08		1.00		0.92		0.95		0.91		
PROMIS-Anxiety	Mean	9.27		8.31		7.38	0.022	8.64		6.96		5.54	0.002	0.131
	SE	2.59		2.35		2.08		2.42		1.7		1.56		
PROMIS-Depression	Mean	7.58		7.35		6.06	0.057	6.72		6.40		6.04	0.274	0.576
	SE	2.24		2.18		1.81		1.99		1.89		1.76		
PROMIS-Fatigue	Mean	13.16		10.72		10.12	0.003	12.98		10.20		9.41	0.001	0.796
	SE	3.08		2.53		2.40		3.04		2.40		2.22		
PROMIS-Sleep disturbance	Mean	14.80		11.52		11.86	0.001	14.62		11.10		10.21	<0.001	0.133
	SE	3.16		2.49		2.56		3.13		2.40		2.20		
PROMIS-Social	Mean	10.20		7.94		7.74	0.017	10.50		8.19		7.33	0.007	0.743
	SE	3.10		2.45		2.39		3.19		2.52		2.26		

Results (estimated means) are generated from generalized mixed-effects models adjusted for age, sex, and BMI.

a P-values are generated from repeated measures generalized mixed-effects models adjusted for age, sex, and BMI (time effects baseline and week 8).

b P-values are generated from repeated measures generalized mixed-effects models adjusted for age, sex, and BMI (time x group interaction).

#### Secondary outcome measures

##### Profile of mood states, abbreviated version total mood disturbance score

As demonstrated in Table 2, there was no statistically significant between-group difference in change in the POMS-A total mood disturbance score over time (*p* = 0.695).

##### Athens insomnia scale

As demonstrated in Table 2, there was a statistically-significant time x group interaction in AIS scores over time (*p* = 0.025). In the Ocimum group, there was a statistically significant 48% decrease in AIS scores from baseline to week 8 (*p* = 0.010) compared to a statistically significant 27% decrease in the placebo group (*p* = 0.028). Moreover, from baseline to week 8, there was a statistically significant greater reduction in AIS scores in the Ocimum group compared to the placebo group (*p* = 0.030). In the Ocimum group, AIS scores reduced by an average of 5.42 points (95% CI, 1.30–9.55, *p* = 0.010) and in the placebo group scores reduced by an average of 3.23 points (95% CI, 0.36–6.11, *p* = 0.028). Pairwise contrasts revealed that the between-group differences in change in AIS scores reached statistical significance at week 8 (*p* = 0.030).

##### Restorative sleep questionnaire, weekly version

As demonstrated in Table 2, there was no statistically-significant between-group difference in change in the RSQ-W score over time (*p* = 0.984).

##### Patient-reported outcomes measurement information system

As demonstrated in Table 2, there was no statistically-significant between-group difference in change in any PROMIS subscale scores over time.

##### Sleep changes as measured by the fitbit

As demonstrated in Table 3, there were no statistically-significant between-group differences in change in total sleep time (*p* = 0.971) or time in bed (*p* = 0.816). In the Ocimum group, there was a statistically significant 3.4% increase in sleep efficiency over time (p = 0.002) and a non-significant change in the placebo group (*p* = 0.268). However, between-group changes in sleep efficiency were not statistically significant (*p* = 0.516).

**Table 3:** Change in wearable sleep tracker measures.

		**Placebo (*n* = 39)**									***Ocimum tenuiflorum*** **(*n* = 44)**									***p*** **-value^b^**
		**Week 1**	**Week 2**	**Week 3**	**Week 4**	**Week 5**	**Week 6**	**Week 7**	**Week 8**	**p-value^a^**	**Week 1**	**Week 2**	**Week 3**	**Week 4**	**Week 5**	**Week 6**	**Week 7**	**Week 8**	***p*** **-value^a^**	
Total sleep	Mean	412.91	412.78	410.96	408.02	407.11	406.79	413.6	414.33	0.895	412.42	427.53	416.44	416.46	414.94	416.98	427.76	425.87	0.175	0.971
time (min)	SE	31.45	31.33	31.14	30.92	31.03	31.08	31.35	31.69		31.28	32.33	31.43	31.43	31.45	31.63	32.28	32.35		
Sleep	Mean	87.38	89.08	86.64	87.39	87.51	87.94	88.15	88.73	0.268	87.23	86.57	86.57	86.95	87.25	86.89	87.74	90.63	0.002	0.516
efficiency (%)	SE	2.86	3.02	2.82	2.83	2.84	2.87	2.88	3.02		2.85	2.91	2.81	2.81	2.82	2.83	2.86	3.05		
Time in	Mean	474.4	470.12	476.25	469.56	466.17	463.33	471.25	468.35	0.640	474.38	495.85	482.21	480.00	476.80	482.91	490.03	478.16	0.747	0.816
bed (min)	SE	37.88	37.48	37.96	37.5	37.37	37.18	37.66	37.79		37.73	39.39	38.28	38.17	38.01	38.49	38.97	38.29		

Results (estimated means) are generated from generalized mixed-effects models adjusted for age, sex, and BMI.

a P-values are generated from repeated measures generalized mixed-effects models adjusted for age, sex, BMI (time effects baseline and week 8).

b P-values are generated from repeated measures generalized mixed-effects models adjusted for age, sex, and BMI (time x group interaction).

##### Maastricht acute stress test

**Figure 3:**
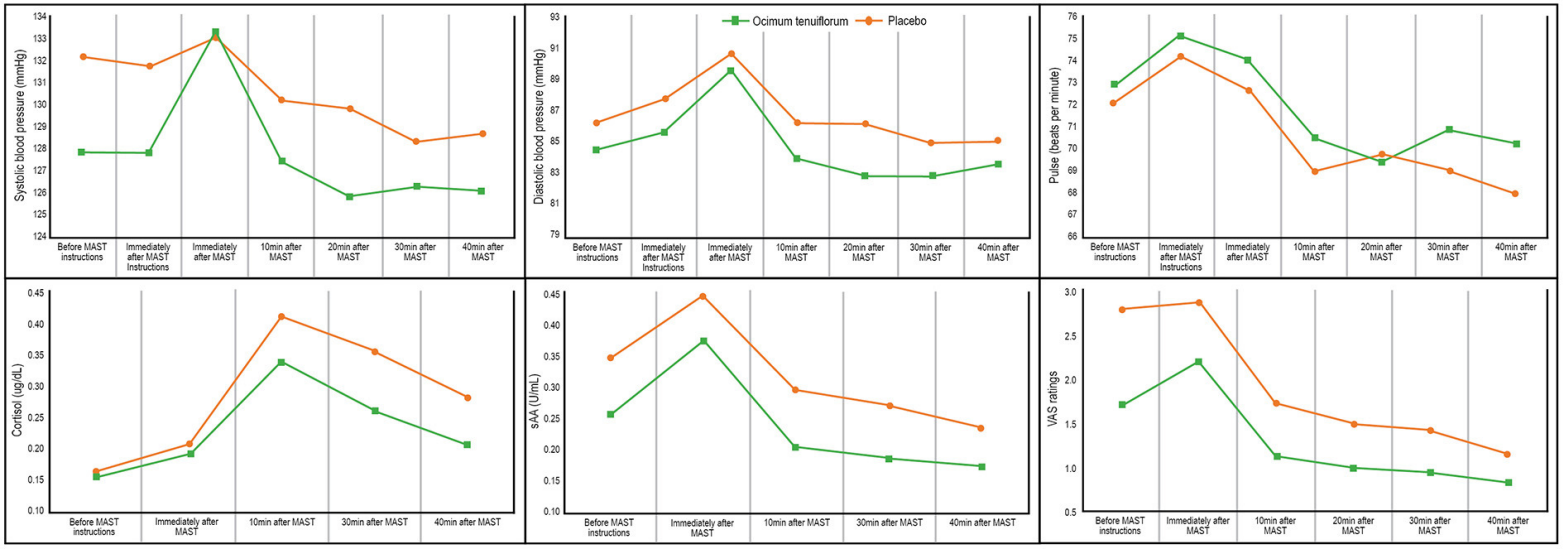
Change in MAST measures.

Changes in blood pressure, salivary cortisol, salivary sAA, pulse, and VAS ratings are detailed in Figure 3 and Table 4. Results from the GLMM revealed there were statistically-significant between-group differences in systolic blood pressure (*p* = 0.010), diastolic blood pressure (*p* = 0.025), VAS ratings (*p* < 0.001), salivary cortisol (*p* = 0.001), and salivary sAA (*p* = 0.001), but not pulse rate (*p* = 0.224). However, there were no statistically-significant time x group interaction effects on any measures.

**Table 4:** Change in MAST measures.

			**Before MAST Instructions**	**Immediately after MAST Instructions**	**Immediately after MAST**	**10 min after MAST**	**20 min after MAST**	**30 min after MAST**	**40 min after MAST**	***p*** **-value (group)**	***p*****-value** **(time x group)**
Systolic blood pressure	Placebo	Mean	132.16	131.78	132.94	130.25	129.82	128.33	128.72	0.010	0.942
(mm Hg)	(*n* = 38)	SE	2.05	2.24	2.38	2.03	1.93	1.90	1.93		
	Ocimum	Mean	127.85	127.82	133.33	127.46	125.84	126.31	126.09		
	(*n =* 43)	SE	1.90	2.07	2.25	1.89	1.78	1.78	1.80		
Diastolic blood pressure	Placebo	Mean	86.24	87.75	90.54	86.22	86.13	84.91	85.10	0.025	0.996
(mm Hg)	(*n =* 38)	SE	1.45	1.70	1.74	1.70	1.68	1.92	1.73		
	Ocimum	Mean	84.57	85.63	89.52	83.97	82.86	82.82	83.64		
	(*n =* 43)	SE	1.36	1.59	1.62	1.58	1.54	1.79	1.62		
Pulse (beats per minute)	Placebo	Mean	72.05	74.21	72.66	68.95	69.75	69.03	67.91	0.224	0.993
	(*n =* 38)	SE	1.95	2.05	2.09	1.85	1.75	1.80	1.65		
	Ocimum	Mean	72.84	75.14	74.06	70.54	69.39	70.84	70.20		
	(*n =* 43)	SE	1.89	1.98	2.03	1.80	1.66	1.77	1.63		
VAS ratings	Placebo	Mean	2.81	-	2.90	1.74	1.51	1.43	1.15	<0.001	0.749
	(*n =* 38)	SE	1.18	-	1.18	1.16	1.16	1.16	1.15		
	Ocimum	Mean	1.73	-	2.22	1.13	1.00	0.95	0.84		
	(*n =* 43)	SE	1.17	-	1.18	1.16	1.16	1.15	1.15		
Cortisol (ug/dL)	Placebo	Mean	0.16	-	0.21	0.42	-	0.36	0.29	0.001	0.221
	(*n =* 38)	SE	0.12	-	0.12	0.12	-	0.12	0.12		
	Ocimum	Mean	0.16	-	0.19	0.34	-	0.26	0.21		
	(*n =* 43)	SE	0.12	-	0.12	0.12	-	0.12	0.12		
sAA (U/mL)	Placebo	Mean	136.92	-	164.81	122.14	-	115.58	105.18	0.001	0.977
	(*n =* 38)	SE	64.73	-	78.01	57.91	-	55.17	50.06		
	Ocimum	Mean	111.55	-	144.16	96.19	-	91.22	87.43		
	(*n =* 43)	SE	52.64	-	68.10	45.51	-	43.41	41.50		

Results (estimated means) and p-values are generated from generalized linear mixed-effects models adjusted for age, sex, and BMI.

##### Hair cortisol

An analysis of cortisol concentrations in newly-acquired hair (0 to 2 cms from the scalp) revealed there was a statistically significant lower concentration of hair cortisol in the Ocimum group (x = 269.68 pg/50 mg; 95% CI 162.61–447.26) compared to the placebo group (x = 789.89 pg/50 mg; 95% CI 228.53–1407.93) (*p* = 0.03).

#### Intake of supplements

Capsule bottles with remaining capsules were returned on the week-8 assessment and a daily medication monitoring phone application was completed by participants. Based on these details, 94% of participants who completed the study took more than 80% of their capsules.

#### Efficacy of participant blinding

To assess the effectiveness of condition concealment during the trial, participants predicted at the end of the study their condition allocation (i.e., placebo, Ocimum, or unsure). Overall group concealment was high as 65% of participants were either unsure or incorrectly guessed treatment allocation (76% of participants in the Ocimum group and 51% of participants in the placebo group). Group differences in the accuracy of treatment prediction were not statistically significant (*p* = 0.072).

#### Adverse events

The frequency of self-reported adverse effects is included in Table 5. No serious adverse events were reported by participants and the frequency of adverse effects was similar in both groups. Two participants withdrew from the study due to self-reported adverse effects associated with capsule intake. One person in the Ocimum group withdrew due to increased agitation, and one in the placebo group withdrew due to ongoing nausea. There were no reports of any adverse events in 83% of participants.

**Table 5:** Frequency of self-reported adverse events.

	**Placebo**	***Ocimum tenuiflorum***
Digestive disturbances	5	5
Headache	2	3
Vivid dreams	1	1
Skin rash	1	1
Sleep disturbances	1	
Agitation		1
Reduced libido		1
Total number of adverse effects	10	12

## Discussion

In this 8-week randomized, double-blind, placebo-controlled study, the administration of an *Ocimum tenuiflorum* extract (Holixer^TM^) was associated with significantly-greater improvements in PSS and AIS scores compared to the placebo, indicating its anti-stress and sleep-enhancing benefits. Based on the PSS, there was a 37% decrease in perceived stress after 8 weeks of intervention in participants taking *Ocimum tenuiflorum* compared to a 19% decrease in people taking the placebo. AIS scores decreased by 48% in the Ocimum group compared to a 27% improvement in the placebo group. Based on data collected from a wrist-worn sleep tracker (Fitbit), Ocimum intake was associated with a 3.4% increase in sleep efficiency over time (compared to a non-significant 0.77% increase in the placebo group). However, between-group changes in sleep efficiency and total sleep time as measured by the Fitbit were not significantly different. Moreover, there were no other statistically-significant between-group differences in changes in the other self-report measures comprising the POMS-A, RSQ-W, and PROMIS. Ocimum supplementation was well tolerated, with no major adverse events reported. Moreover, due to the infrequency of self-reported adverse events, and no significant between-group differences in the frequency of self-reported symptoms, it is unlikely that the adverse events can be attributed to the intake of *Ocimum tenuiflorum*.

Inconsistencies in the self-report measures may be due to differences in the time intervals utilized by the measures as the AIS and PSS required respondents to provide ratings over the last 2 and 4 weeks, respectively. In contrast, shorter time intervals were used in the PROMIS-29 and RSQ-W (7 days) and POMS-A (right now). There were trends to suggest better improvements in the PROMIS anxiety and sleep disturbance subscales in the Ocimum compared to the placebo group. Therefore, it is possible that the PROMIS may be less sensitive to change than the PSS and AIS, which are self-report measures specifically designed to measure perceived stress and insomnia. Further research is required to help elucidate the reasons for the discrepancies in these findings. The non-significant between-group differences in Fitbit sleep measures may be due to weaknesses in the accuracy of Fitbit recordings. Fitbit devices are comparable to data collected by actigraphy and sleep diaries but tend to overestimate total sleep time and sleep efficiency compared to PSG (19–21). The use of more accurate and validated sleep measures such as actigraphy, PSG, and electroencephalogram will be helpful in future trials. Differences in the self-report and objective sleep measures may also be associated with different cognitive and somatic constructs of the pathophysiology of insomnia. More specifically, subjective insomnia severity may be associated positively with sleep effort, whereas objective sleep measures such as PSG may be more strongly associated with pre-sleep arousal and dysfunctional beliefs and attitudes about sleep (29). It is also important to note that it was hypothesized that chronic *Ocimum tenuiflorum* intake would be necessary for sleep-enhancing effects to occur. Therefore, no baseline data were collected before the administration of *Ocimum tenuiflorum*. In future trials, it would be helpful to collect 1 to 2 weeks of sleep data before *Ocimum tenuiflorum* intake.

To help identify the effect of *Ocimum tenuiflorum* on the stress response, participants were exposed to an acute experimental stress procedure (MAST), and hair samples were collected to examine between-group differences in chronic cortisol excretion. The results from the MAST indicated that *Ocimum tenuiflorum* buffered physiological and subjective responses to acute stress as evidenced by significantly lower concentrations in salivary cortisol, sAA, blood pressure, and subjective stress ratings compared to people taking the placebo. The reduction in salivary sAA and cortisol suggests that Ocimum has a buffering effect on the SAM system and the HPA axis, respectively, in response to an acute stressor. Hair cortisol concentrations were also significantly lower in participants taking *Ocimum tenuiflorum* which suggests Ocimum can dampen HPA-axis activity in the long-term. *Ocimum tenuiflorum* was well tolerated, with no significant adverse effects reported by participants.

Even though *Ocimum tenuiflorum* has a long history of traditional use as an anti-stress agent, clinical trials are limited. In an open-label, 60-day trial on adults with generalized anxiety disorder, 500 mg, twice daily delivery of an *Ocimum tenuiflorum* leaf extract was associated with significant improvements in anxiety, stress, and mood; however, this study was flawed due to its no placebo control (10). In a randomized, placebo-controlled, 6-week trial on adults experiencing symptoms of stress, 1,200 mg daily of an *Ocimum tenuiflorum* extract (OciBest^®^) reduced ratings of forgetfulness, sexual problems, feelings of exhaustion, and sleep problems more than the placebo (9). The results obtained from the current trial add to the body of evidence supporting the efficacy of *Ocimum tenuiflorum* as an anti-stress agent in adults experiencing stress and poor sleep. However, in this trial, anti-stress and sleep-enhancing effects occurred at a lower dose of 250 mg daily, compared to the previous trials where doses of 600 to 1,200 mg daily were administered.

The anxiolytic and antidepressant effects of *Ocimum tenuiflorum* have been identified *via* previously conducted animal stress and depression studies such as the forced swim test, tail suspension test, and acute noise stress trials (7, 30–32). These beneficial effects may be due to *Ocimum tenuiflorum* influencing activity in HPA-axis and SAM system as this plant, along with its constituents such as ocimumosides and ocimarin, inhibited corticosterone release, protected against adrenal hypertrophy, acted as a corticotropin-releasing hormone receptor 1 (CRHR1) antagonist, and inhibited 11β-hdroxysteroid dehydrogenase (11β-HSD1) and catechol-O-methyltransferase (COMT) activity (7, 30, 33). 11β-HSD1 is an enzyme involved in the conversion of inactive cortisone into cortisol, and COMT is an enzyme involved in the catabolism of monoamines. Other mechanisms of action potentially associated with the anti-stress effects of *Ocimum tenuiflorum* include its ability to reduce stress-induced alterations in monoamines such as serotonin and dopamine, and markers of oxidative stress (34, 35).

The results of this trial suggest that *Ocimum tenuiflorum* can reduce HPA-axis activity and the sympathetic response after exposure to an acute stressor. This was evidenced by overall reductions in cortisol, sAA, blood pressure and stress ratings before and up to 40 min following exposure to the MAST. The MAST is a 12 min experimental procedure involving physical and mental stress where participants were required to immerse their hand in extremely cold water and engage in a demanding mental arithmetic task. *Ocimum tenuiflorum* had an overall stress-buffering effect both before and after this stress procedure. Further evidence of the impact of *Ocimum tenuiflorum* on HPA-axis activity was demonstrated by significantly lower hair cortisol concentrations in people taking Ocimum for 8 weeks compared to the placebo. Hair cortisol provided a chronic measure of cortisol excretion, suggesting *Ocimum tenuiflorum* taken for 8 weeks can reduce overall cortisol output in adults experiencing stress and poor sleep.

### Study strengths, limitations and directions for future research

The primary strength of this study includes the use of both objective and subjective outcome measures to assess the stress-relieving and sleep-enhancing effect of *Ocimum tenuiflorum* in adults experiencing high stress. Moreover, the inclusion of an experimental stress procedure (MAST) helped demonstrate the impact of *Ocimum tenuiflorum* in adults exposed to a moderately aversive physical and mental stressor. However, despite several positive findings from this study, there are several limitations and suggested directions for future research. In this study, a hair sample was collected at week 8. Because it has been commonly reported that hair grows approximately 1 cm per month (36), it was expected that 2 cm of hair closest to the scalp would provide an estimate of cortisol output over the previous 8 weeks (i.e., during the study period) while hair 2 to 4 cm away from the scalp would provide an estimate of cortisol output 8 to 16 weeks ago (i.e., 0 to 8 weeks before the commencement of capsules). However, the substantial between-group differences in hair cortisol measurements 2 to 4 cm away from the scalp suggested this may not have been the case. A review of the literature confirms that the commonly proposed mean hair growth of 1 cm per month may be an oversimplification as not all hair grows at any one time, and the percentage of follicles in a growth phase is influenced by the anatomical location, age, sex, and race (26–28). To investigate changes in hair cortisol over time, it will be preferable in future trials to collect hair samples both at baseline and end of treatment so that a more reliable measure of change in cortisol can be undertaken. Finally, future research will be required to help identify the efficacy of different *Ocimum tenuiflorum* extracts, delivered at varying doses and treatment periods. Moreover, identifying its benefits based on age, sex, in people experiencing varying environmental and social stressors, and in people diagnosed with anxiety-related disorders will be important.

In conclusion, the results from this 8-week, randomized, double-blind, placebo-controlled trial revealed that *Ocimum tenuiflorum* supplementation in stressed adults experiencing poor sleep was associated with improvements in self-report measures of perceived stress and sleep quality. *Ocimum tenuiflorum* also reduced the stress response after exposure to an acute stressor and reduced chronic cortisol excretion as measured by hair cortisol concentrations. There was a statistically significant 3.4% increase in sleep quality in participants taking Ocimum, as measured by a wrist-worn sleep tracker; however, between-group changes in sleep efficiency and total sleep time did not reach statistical significance. Moreover, there were no changes in other utilized self-reported mood measures. Future trials using varying treatment periods, gold-standard sleep monitors, and different populations will be important to confirm and extend upon the findings from this study.

## Data availability statement

The raw data supporting the conclusions of this article will be made available by the authors, without undue reservation.

## Ethics statement

The studies involving human participants were reviewed and approved by National Institute of Integrative Medicine. The patients/participants provided their written informed consent to participate in this study.

## Author contributions

AL and SS designed the research and conducted the research. AL, PD, and AM analyzed the data. AL, PD, and SS wrote the paper. All authors read and approved the final manuscript.

## Funding

The study received funding from Natural Remedies Pty Ltd. The funder was not involved in data collection, analysis, interpretation of data, writing of the manuscript, or the decision to submit it for publication.

## Conflict of interest

This study received funding from Natural Remedies Pty Ltd. The funder was not involved in the design of the research study, analysis of data, or writing of the report. AL was the managing director of Clinical Research Australia, a contract research organization that has received research funding from nutraceutical companies, also received presentation honoraria from nutraceutical companies. SS was an employee of Clinical Research Australia. The remaining authors declare that the research was conducted in the absence of any commercial or financial relationships that could be construed as a potential conflict of interest.

## Publisher's note

All claims expressed in this article are solely those of the authors and do not necessarily represent those of their affiliated organizations, or those of the publisher, the editors and the reviewers. Any product that may be evaluated in this article, or claim that may be made by its manufacturer, is not guaranteed or endorsed by the publisher.
